# Life Insurance

**Published:** 1859-03

**Authors:** 


### HALL'S JOURNAL OF HEALTH.

OUR LEGITIMATE SCOPE IS ALMOST BOUNDLESS: FOR WHATEVER BEGETS PLEASURABLE AND HARMLESS FEELINGS, PROMOTES HEALTH; AND WHATEVER INDUCES DISAGREEABLE SENSATIONS, ENGENDÈRS DISEASE.

*We aim to show how Disease may be avoided, and that it is best, when sickness comes, to take no Medicine without consulting an educated Physician.*

VOL. VI.] MARCH, 1859. [No. 3.

### LIFE INSURANCE.

WE consider it unbecoming a philosopher and a christian, to have anything to do with these establishments, directly or indirectly. Faro bank dealers, lottery men and stock jobbers are tumbled over into the hands of the "adversary," to be dealt with *secundem artem*, without the slightest compunction, and every where there is a repugnance against the failure of a fair *quid pro quo:* while religious men are so horrified at any thing like "chance," that they won't "draw straws!" The gambler says "heads, I loose, tails, you win;" the insurance company says, in effect, "I will bet you two thousand dollars, against fifty-six dollars, that you won't die in a year, provided you pay the fifty-six dollars in hand, and take our word for the payment of the two thousand, in case you should die." It seems to us that there is a slight degree of downright impertinence in the "transaction," with no small share of impiety in the phrase "I will insure your life for one year!" If the insured dies within the year, his family or friends receive two thousand dollars, for fifty-six; there is no equity in that—no just reciprocity. Many a lottery policy will give you a chance of getting five times as much money for one-tenth of the amount. There ought to be a repugnance in the mind of a husband or wife, or other near relation, against reaping a benefit by virtue of the death of the other party: therefore, we say to every Christian man, "have faith in God," that the experience of the sweet singer of Israel will be fulfilled in your children—"I have been young, and now I am old, yet have I not seen the righteous forsaken, nor his seed begging bread." Besides this, the most desponding of all the prophetical writers enjoins,—"leave thy fatherless children, I will preserve them alive; and let thy widows trust in me." To our mind these things mean something, they mean a great deal; they mean all that they say. God fulfils his promises literally, giving "good measure, pressed down, and shaken together, and running over." The barrel of meal decreased not, neither did the cruse of oil fail in old Elijah's day, nor will it ever, to the truly trusting, unless for higher advantages.

But the Almighty's ways are the best ways, even in a pecuniary point of view, for they not only habituate the mind to humble trustingness, they are profitable, both as to the life that now is, and that which is to come. Let us look at the life insurance figures. They make money by it; the daily papers show that they are dividing ten and twenty per cent. annually, and these immense dividends are profits paid by the poor and the struggling, to those who are clothed in purple and fine linen, and fare sumptuously every day; who toil not, neither do they spin, but lounge on velvet cushions, and roll along our streets in equipages, vieing in splendor with those of princes, which they have a sort of right to do, if the daily toiler chooses to pay the piper.

Insurance companies are very clear of giving policies to any but persons in sound health; and that the chances of life are in favor of the company, the profits at each annual report clearly show. The best insurance is a temperate, rational life, with the immense advantage, that the insured in this case, lives to enjoy his policy instead of its being done by the husband of his widow.

Then again, policies are frequently paid only at the end of a law suit. So common is this, that it is not an unusual thing, when a prominent person dies, and his family connection is too powerful for a company to contend with, the policy is promptly paid with a flourish of trumpets in the daily papers, which catches gulls, as the announcement of the drawing of the highest prize in a lottery leads multitudes to try their chance, which otherwise they never would have thought of doing.

There is another item, the presidency and secretaryship and brokerage of an insurance company are valuable births, each worth thousands of dollars, some of them five thousand dollars a year; and yet, after all these salaries are paid, annual dividends are made, reaching to twenty per cent.—who pays all this? Poor clergymen, from meagre salaries, eked out by painfnl economies; working men, whose daily labor procures that same day's need; loving parents and self-denying husbands, who seldom part with a premium without a pang.—Shame on the whole thing!

But there are cases not a few, where honorable men have paid the premiums for many years, amounting to thousands of dollars, when the company "fails;" and being now too old to secure a fresh policy in some other office, except at a price which is simply impossible to them, they, in a year or two die, with an amount of money in the company's strong box, which would make their helpless families comfortable for years. If then, a man wants to lay up a safe treasure for his family in case of his death, we propose to him a plan for securing his life, and the premiums of a life time too. As to the first we say, live in temperance, moderation, cleanliness, and then lay by a premium every year, to be put out at legal interest, payable quarterly, which also put out in the same way, and in the course of twenty years, the amount on hand would be greater than the amount received from the company would be, and just double in thirty years. So if the insurer makes money, the insured can make money too, by insuring himself.

Be it remembered, that insurance companies require a man to be in good health, of regular habits, and to avoid hazardous occupations; then, for fifty-six dollars, at forty-one years of age, they will insure his life for one year for two thousand dollars; but a man at forty-one, of such a character as the above, stands a good chance of living to seventy years, the three score and ten of scripture; in that case, his family will receive at his death, just half what he has paid, but double that if he had insured himself.

We say, therefore, to every christian reader, keep away from the life insurance office, whose foundation is on chance; as is the lottery, the faro bank, and the stock board. On the other hand, have faith in God, in a regular temperate life and in a true economy; living within your income, and resolutely, with an invincible determination, put the surplus at safe interest and collect it quarterly. Doing this from year to year is true wisdom, for it is a more profitable investment than any life insurance company dares to offer. Scarcely had we closed the last paragraph, when carriage after carriage rolled along the street, and above the clatter of wheels was heard glad sounds of women's voices, and men speaking quick and cheerily, for they had all brought up next door, on the occasion of a complimentary visit to an aged clergyman, who for fifty-eight years had been an active, eloquent and an efficient minister in the methodist church; and still, in his keen black eye is the fire of younger years, for Sabbath after Sabbath he fails not to preach the gospel. But one just closing his eightieth year needed a staff, and a handsome one they gave him; rather heavy, however, for they had "made a deposit" within it of four hundred five dollar gold pieces, which was so unexpected to him, that he could only say that he regarded it as another evidence of the goodness of that Providence which had never failed him in his past pilgrimage, and would not fail him unto death. Had it been at all known that such a manifestation was in progress, there are multitudes in New York of all denominations, who would have considered it a pleasure and a privilege to have been allowed to participate in gladdening the aged heart of so useful and good a man as NATHAN BANGS, of Irving Place.

After all, who shall not say that the best insurance office is not in Wall street, but in Heaven, where "premiums" are paid, not in gold and silver, not in bills and checks, but in the privilege of a useful and guileless life, a life honored and honorable, for such a life has been that of the revered father whose name we have written.

